# Computational Predictions and Evolutionary Analysis of *LrK10* Kinase-Related Putative *PSTOL1* Gene Homeologs in Wheat and Orthologs of Its Wild Relatives

**DOI:** 10.3390/ijms27104513

**Published:** 2026-05-18

**Authors:** Karthikeyan Thiyagarajan, Kalenahalli Yogendra, Carolina Saint Pierre, Santosh Kumar Singh, Chitranjan Kumar, Doyeli Sanyal, Garima Thakur, Deepika Singh, Deepshikha Thakur, Ajay Tomar, Prashant Vikram, Ravi Valluru

**Affiliations:** 1 Global Wheat Program, International Maize and Wheat Improvement Centre (CIMMYT), El-Batan 56237, Mexico; c.saintpierre@cgiar.org; 2 Amity Institute of Organic Agriculture, Amity University, Noida 201313, Uttar Pradesh, India; ckumar@amity.edu (C.K.); dsanyal@amity.edu (D.S.); gthakur1@amity.edu (G.T.); dsingh21@amity.edu (D.S.); dthakur1@amity.edu (D.T.); atomar2@amity.edu (A.T.); 3 School of Agriculture, Food and Wine, The University of Adelaide, Adelaide, SA 5064, Australia; yogendra.kalenahalli@icrisat.org; 4 International Crops Research Institute for the Semi-Arid Tropics, Patancheru, Hyderabad 502324, Telangana, India; 5 Morehouse School of Medicine, Atlanta, GA 30310, USA; sksingh@msm.edu; 6 Indian Agricultural Research Institute, New Delhi 110012, Delhi, India; pvikramseedwheat@gmail.com; 7 Lincoln Institute for Agri-Food Technology, University of Lincoln, Lincoln LN6 7TS, UK

**Keywords:** *TaLrK10*, *TaPSTOL1*, *Poaceae*, *Triticeae*, wheat, wild relatives

## Abstract

*Phosphorus Starvation Tolerance 1* in rice (*OsPSTOL1*, known as *Phosphorus uptake 1*, *Pup1*) is a receptor-like cytoplasmic protein kinase that confers tolerance to phosphorus deficiency. The *OsPSTOL1* gene possesses a Ser/Thr kinase and shows high amino-acid sequence similarity with the *leaf rust receptor-like kinase* (*OsLrK10*). We hypothesise that the putative wheat genes *TaPSTOL1* and *TaLrK10* have a common ancestral origin and that putative *TaPSTOL1* diverged recently, acquiring new structural modifications and biological functions in the process. In this study, we identified all putative *TaPSTOL1* homeologs and examined the evolutionary relationship between *TaPSTOL1* and *TaLrK10* in Triticum species. Our results indicate that the putative *TaPSTOL1* diverged recently without possessing the amino-terminal domain, which is a typical characteristic of *TaLrK10*. We observed numerous conversion tracts between these two genes, and the substitution pattern of randomly selected amino acids indicates that dynamic selection pressures acted on both genes. The putative *TaPSTOL1* shows high nucleotide diversity compared to *TaLrK10* within *Triticum* species. Further, a multiple-sequence analysis reveals that the third exon of *TaLrK10* appears to have been duplicated and diverged as a putative single-exon-based *TaPSTOL1* in bread wheat. Overall, our comparative analysis indicates that both *TaPSTOL1* and *TaLrK10* appear to have diverged from a common ancestor, acquiring distinct structural organisations and biological functions.

## 1. Introduction

Phosphorus (P) is an essential macronutrient constituting approximately 0.2% of the total dry weight in plants [1]. It is involved in many vital processes such as photosynthesis [2], lipid metabolism [3], nucleic acid synthesis [4] and signalling [5]. However, due to the low levels of bioavailability and its slow diffusion in the soil (diffusion coefficient of 10^−7^ to 10^−9^ cm^2^ s^−1^), P uptake is a constraint for the plant [6,7]. It is therefore essential to identify and understand the genetic mechanisms regulating the absorption and utilisation of P availability.

A genetic locus conferring P-deficiency tolerance (*Phosphorus uptake 1*, *Pup1*) was previously identified in an *aus*-type Indian rice variety, Kasalath [8]. Near-isogenic lines with the *Pup1* locus harbouring Kasalath allele showed 3-fold higher P-uptake and grain yield under low-P conditions [8]. Subsequently, a gene encoding Ser/Thr kinase of the Receptor-like Protein Kinase LRK10L-2 subfamily from the *Pup1* locus, *Phosphorus Starvation Tolerance* 1 (*OsPSTOL1*), was identified as the most likely gene responsible for the phenotype [9]. *OsPSTOL1* is a receptor-like cytoplasmic protein kinase (*RLCK*) that shows phosphorylation activity. In plants, about 1–3% of the total number of genes encode for putative proteins showing kinase activity that is involved in many metabolic processes [10]. Further, lines overexpressing *OsPSTOL1* showed approximately 30% higher grain yield in comparison with the null lines [9]. Indeed, studies identified several novel single-nucleotide polymorphisms (SNPs) of *OsPSTOl1* in rice [11,12,13] and haplotypes based on SNPs in wild rice *O. rufipogon* are associated with significant differences in root length and root weight [9].

Subsequently, using *OsPSTOL1* amino-acid sequence as a query, several *PSTOL* homologs were identified in several crop species, including six *SbPSTOL* homologs in sorghum [14] and six *ZmPSTOL* homologs in maize [15]. These identified proteins enhanced plant performance, grain yield and root system architecture traits under low-P and were clustered together with *OsPSTOL1* from rice. Further, these identified proteins indicate that the sequences were conserved mostly in the kinase domain. In wheat, the *TaPSTOL1*-like gene was resequenced (from chromosome 5A) from 27 different bread wheat and durum wheat accessions, characterised the expression of *TaPSTOL1* under different P concentrations and demonstrated the induction of promoter in root tips and root hairs under low-P conditions [16]. These studies emphasise that the *PSTOL1* gene has the potential for molecular breeding applications to improve crop performance under low-P conditions.

The *OsPSTOL1* protein shows a high amino acid sequence similarity with the *leaf rust receptor-like kinase* (*LrK10*), located in the *Leaf Rust Resistance* (*LRR*) locus that belongs to the family of *Receptor-Like Kinases* (*RLKs*) [9,17]. Further, *TaLrK10* is involved in leaf rust disease resistance in bread wheat [18]. Hence, *PSTOL1* and *LrK10* belong to *RLCKs* and *RLKs*, respectively, and both carry a Ser/Thr kinase phosphorylation domain. *RLKs* typically have an amino-terminal domain protruding into the extracellular space, while *RLCKs* lack this extracellular domain [19]. Previously, six copies of *Lrk* genes were identified by Southern hybridisation on the three homeologous group 1 chromosomes in wheat [20,21]. The evolutionary relationships between all of the members of the *Lrk* gene family from the A, B, and D genomes of wheat and its progenitors suggest that *Lrk* copies in hexaploid wheat were phylogenetically sisters to the orthologous copies in the parental diploid species, suggesting that the sequences of the homeologous *Lrk* genes evolved independently after polyploidisation [22].

Interestingly, assessing the paralogous and orthologous relationships among the *Lrk* genes indicated that gene losses have occurred for this gene family in the Triticeae [22]. Two paralogous loci comparisons further highlighted that while *Lrk* gene content and organisation are well conserved, some of the noncoding regions showed a great extent of reshuffling, indicating several local duplications, deletions, and insertions. It has been opined that small duplications of the *Lrk* gene sequences with no repetitive elements have occurred successfully in Triticeae [22].

As both *PSTOL1* and *LrK10* show high amino-acid sequence similarity with each other [9,17], we hypothesised that the putative *PSTOL1* is derived from *LrK* sequences through duplication, which then diverged as a putative *PSTOL1*, acquiring a distinct biological functionality during the process. Here, we identified all the putative *TaPSTOL1* homolog sequences in wheat and examined the evolutionary relationship between putative *TaPSTOL1* and *TaLrK10* in *Poaceae* species, focusing on the tribes of *Triticeae*, *Brachypoideae*, *Paniceae*, and *Andropogoneae*. Our results support the hypothesis showing that the putative *TaPSTOL1* has a high nucleotide identity (96%) with the third exon of *TaLrK10* in wheat.

## 2. Results

### 2.1. Bread Wheat Has Eight Putative TaPSTOL1-like Gene Sequences

**Figure 1:**
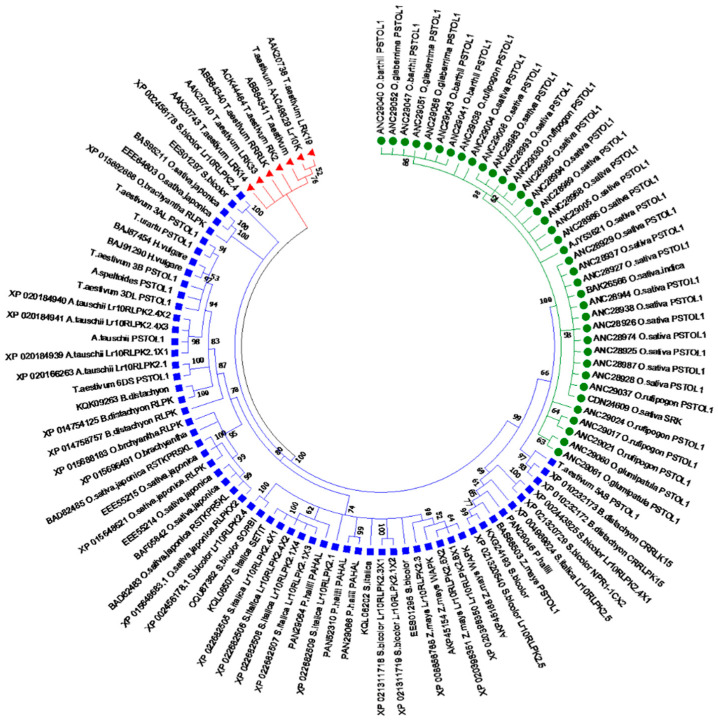
Phylogenetic tree based on the protein sequences of PSTOL1 and LrK10 in diverse Poaceae members. Clade I: Round green shape symbol: Oryza species consists of PSTOL1 and receptor-like kinase, Clade II: Blue Square Shape: Triticeae and other Poaceae members with PSTOL1 and Receptor kinase genes, Clade III: Red Triangle shape: Triticum aestivum receptor-like kinases.

Using the rice *OsPSTOL1* amino acid sequence as a query (Accession: KU922625) [23], we identified nine wheat sequences with high similarity to *OsPSTOL1*. We observed the first hit on chromosome 5A (E-value 0.0, [16]) and subsequent hits on group 3 chromosomes (3A, 3B, and 3D with E-value ranging from 2 × 10^−139^ to 7 × 10^−138^). Due to close sequence similarity with *OsPSTOL1* (Figure S1) and recent natural hybridization of the D genome into hexaploid wheat, we further chose the 3DL homeolog sequence as a query to search for homologs/orthologs in closely related species and wheat progenitors. We identified eight putative *TaPSTOL1* homeolog sequences in the bread wheat cultivar Chinese Spring that share 96% identity at the amino acid level. These homeologs are present on chromosomes 3AL, 3B, 3DL, 5AS, 6DS, 7AL, 7BL, and 7DL with an identity ranging from 68% to 95% (Table S1). We further identified five putative paralogs in durum wheat with nucleotide identities ranging from 67 to 95%. Four putative paralogs were identified in wheat progenitors *T. urartu* (67–95% identity) and *A. speltoides* (65–95% identity), and two putative paralogs in *A. tauschii* (68–96% identity). Similar to *OsPSTOL1*, all eight putative wheat *TaPSTOL1-like* sequences identified are predicted to have a conserved Ser/Thr kinase domain that is conserved across all phyla (Figure 1). In order to further confirm the evolutionary relationship between the *PSTOL1* gene and the *LrK10*, the *PSTOL1* gene 3DL homeolog was subjected to BLASTN analysis and found several top hits in numerous *Triticeae* species. Therefore, the top hit with e-value 0.0 and 97% query coverage was further used (XM_044501583.1) against the Ensembl wheat BLASTN and found specific *LrK10* candidate genes (TraesCS3D02G261800, TraesCS3B02G295000, TraesCS3A02G261800). It is not surprising that both have common ancestry and physical locations in bread wheat; however, activity to phosphate deficiency and immunity make them express as different genes with unique locations on the genome driven by unique promoters, as evidenced through Figure S2b with the predicted promoter transcription factor binding motifs in the upstream region (−1200 bp) for transcripts of *AtLrK10* and *TaLrK10* (Chromosome 3D).

**Figure 2:**
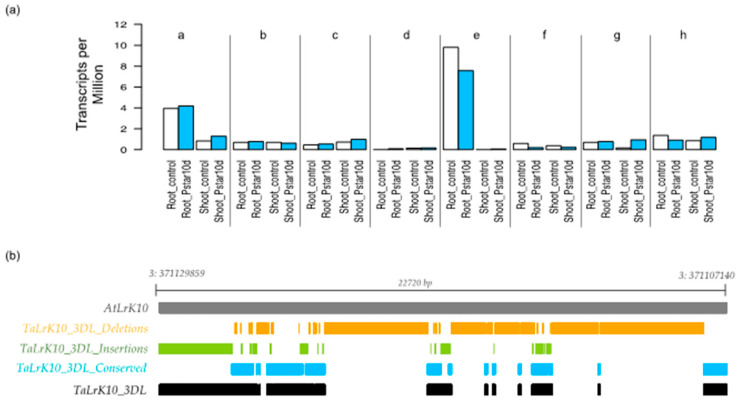
(**a**) Expression of putative *TaPSTOL1*-like homeologs grown under control (0 days) and P-starvation (10 days) in Chinese spring ([24]; a–h: Traes_3AL_B27A64367, Traes_3B_34896ED29, Traes_3DL_E468838BA, Traes_5AS_841ECFF18, Traes_6DS_84B6888, Traes_7AL_F046FDB9E, Traes_7BL_504698D18, and Traes_7DL_E183F108C, respectively). (**b**) Comparison of nucleotide differences between putative *AtLrK10* and *TaLrK10.* Considering the *AtLrK10* as an ancestral gene, we identified and plotted both INDELs and conserved nucleotides in *TaLrK10*_3DL in bread wheat. At, *Aegilops tauschii*; Ta, *Triticum aestivum*; 3DL, chromosome 3DL; Pstar10d, P-starvation for 10 days.

The gene expression profiles of putative *TaPSTOL1-like* were queried in Chinese spring wheat grown under P-starvation for 10 days by probing the wheat expression database (Accessed on 23 February 2019 www.wheat-expression.com), queried on April 13th, 2019; was already published [24]. In general, all homeologs of putative *TaPSTOL1-like* were more expressed under low-P as compared to the controls (Figure 2a). A tissue-specific expression pattern was observed for all homeologs in response to 10-d P-starvation (Figure 2b), suggesting that all the putative *TaPSTOL1-like* homeologs identified in the study are expressed under low-P conditions.

We further identified putative *LrK10* sequences in bread wheat and its wild relatives using a BLASTN (in NCBI) search with the option MEGABLAST. This search revealed a gene model (*Lr10*, *LOC109770626*; hereafter *AtLrK10*; GenBank accession for *mRNA*: XM_020329350) that is likely showing alternative splicing of three transcript variants whereby X1 and X2 have three exons while transcript variant X3 has four exons (Figure S2a). The second exon (from transcript variant X3) appears to have been lacking in transcripts X2 and X1. The third and fourth exons shared the same genomic positions; however, the first exon genomic position varied between three transcript variants (Figure S2a). The presence of *TaLrK10* from bread wheat was previously reported on chromosome 1A [18]. We identified a gene model from IWGSC (*TraesCS3D02G261800*) with exon/intron boundaries harbouring four transcripts (hereafter denoted as *TaLrK10_3DL*). This gene locus harbours four transcripts, among which transcripts one and three appear to have protein-coding, carrying three exons each, while transcripts two and four appear to be nonfunctional (Figure S2a). Overall, the BLASTN searches identified several putative homeologs, paralogs, and orthologs of *TaPSTOL1-like* and *TaLrK10* sequences in wheat and its wild relatives. Further, all putative *TaPSTOL1-like* homeologs expressed more under low-P and were predicted to have numerous transcription factor binding motifs in their upstream region (−1200 bp) of the transcript start site associated with the regulation of RLKs (Figure S2b).

### 2.2. The Third Exon of TaLrK10 as a Putative TaPSTOL1 Gene with a MITE Insertion

To understand the identity between *AtLrK10* and *TaLrK10_3DL*, we further compared both *AtLrK10* and *TaLrK10_3DL*, which revealed that the putative *TaLrK10_3DL* had a 95% identity with the transcript X1 of *AtLrK10*, while the third exon of *AtLrK10* showed an identity of 96% (Figure S3). Indeed, other than the conserved exons, the rest of the regions have shown many INDELs (insertions: 19% and deletions: 71% relative to the *AtLrK10*) were identified in *TaLrK10_3DL*, while 24% of nucleotides appear to have been conserved between *AtLrK10* and *TaLrK10_3DL*. We also compared the putative *TaLrK10_3DL* with the *TaLrK10* gene from chromosome 1A [18], indicating that the *TaLrK10* from chromosome 1A shared 58.49% sequence similarity with *TaLrK10_3DL* and 57.09% sequence similarity with *TaLrK10_3AL*.

We hypothesised that the putative *TaPSTOL1-like* is duplicated and diverged from *TaLrK10*. To understand the commonality between them, we aligned sequences of *TaLrK10_3DL* and putative *TaPSTOL1-3DL* from bread wheat (Figure S3). We noticed that the physical genomic location of *TaLrK10* from both *A. tauschii* and bread wheat overlaps with the putative *TaPSTOL1-like* sequence, suggesting that both sequences are present within the same locus region. Our computational analysis further revealed that the third exonic region of *TaLrK10* (with reference to homeolog from 3D) appears to have duplicated and diverged as the putative *TaPSTOL1-like* gene. Indeed, the third exonic region of *AtLrK10*, *TaLrK10_3DL*, and the putative *TaPSTOL1-3DL* shared an identity of 96% (Figure S3). The other two exons encoding the extracellular N-terminal domain in *TaLrK10* did not show any sequence similarity with *TaPSTOL1-3DL* due to the absence of the N-terminal domain in putative *TaPSTOL1-3DL*. Overall, our analysis suggests that the third exon of *TaLrK10* (location in 3DL: 363,607,922 bp to 363,608,939 bp) could indeed act as the putative single exon-based *TaPSTOL1* gene.

**Figure 3:**
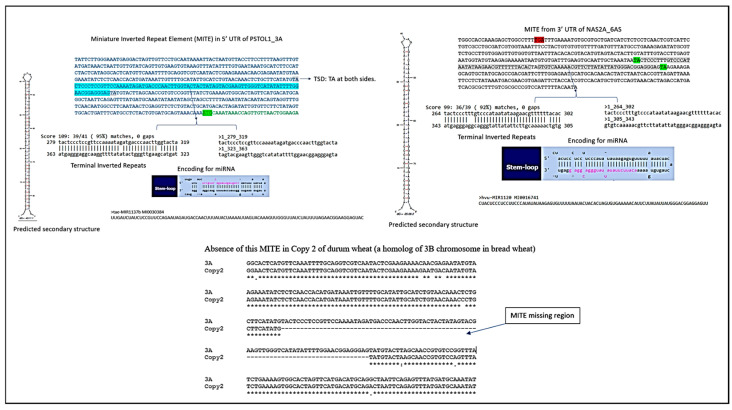
Prediction of MITES and MITES encoding for miRNA from specific homeologs and the comparison of the absence of MITE from a copy2 of durum wheat, with a resemblance to the 3B homeolog of bread wheat, as the insertion is only observed in the 3A homeolog chromosome of bread wheat.

We further observed an insertion of MITE (Miniature Inverted Repeat Transposable Element) in the 5′UTR of putative *TaPSTOL1-like* from chromosome 3AL in bread wheat (approx. 90 bp in length, containing a 42 bp TIR (Terminal Inverted Repeat) and a 2 bp Target Site Duplication (TSD)). This MITE is absent in the 5′UTR region of *TaLrK10* from chromosome 1A, while it has many mutations in putative *TaPSTOL1-3DL* and *TaLrK10_3DL* in bread wheat. The identified MITE encodes for the miRNA tae-MIR1137b MI0030384 (Figure 3), which was previously reported to be highly expressed in mature embryos of wheat during grain development [25,26]. Among wheat progenitors, this MITE is only present in *T.urartu* (Table S2). In tetraploid durum wheat, the available information on only one of the two homeologous copies of this gene suggests that the MITE insertion is present only on chromosome 3A, while presumably absent on chromosome 3B. Consistently, the MITE insertion was observed in the putative *TaPSTOL1* on chromosomes 3A and 3D (with many mutations) but absent on chromosome 3B in bread wheat.

We identified 197 putative target genes for *tae-MIR1137b*, including four protein kinases. However, there was no hit observed for either *TaPSTOL1_3DL* or *TaLrK10_3DL*, probably due to the absence of the MITE. This analysis suggests that this miRNA may have essential roles in the regulation of genes; however, it is unknown whether it regulates putative *TaPSTOL1-like* homeologs. Overall, our comparative analysis suggests that the third exon of *TaLrK10* and putative *TaPSTOL1-like* share high nucleotide sequence similarity in bread wheat. Further, the MITE insertion appears to be present in the A and D sub-genomes, while absent in the B sub-genome of bread wheat. The MITE is intact in a subgenome (3A); hence, it is presumed that the encoding miRNA may affect its target genes; however, the *PSTOL1* gene from 3B or 3D might not have been directly affected by this miRNA. However, the aberrant fragments of miRNA or the transcripts may be involved in gene silencing from 3A to 3D, for instance, due to the partial conservation nature of miRNA-encoding MITE from 3A to 3D.

### 2.3. Phylogenetic and Diversity Analysis Based on the Protein Sequences of Putative TaPSTOL1-like and TaLrK10

The protein-based phylogenetic tree of putative *TaPSTOL1-like* and *TaLrK10* within *Poaceae* species indicates that all species studied with this phylogeny belonged to three clades (Figure 2). The first clade consists of *OsPSTOL1* from *Oryzeae* species alone, suggesting the presence of *Oryzeae* species-specific haplotypes based on these two proteins (Figure S4; e.g., “YKGELPNGVPVAVKMLEN” in *Oryzeae* species versus “YRGSLPNGREIAVKMLKD” in *T. aestivum_Lr10K_* AAC49629_U51330). This clade clustered distantly from the second clade (see below). For instance, *OsPSTOL1* from *O. sativa* and *TaLrK10* from *T. aestivum* have 70% polymorphisms. Hence, *OsPSTOL1* from *Oryzae* species clustered distantly from those of *TaPSTOL1*/*TaLrK10* (clade II) of other tribes of *Poaceae* species.

The protein-based phylogenetic tree of putative *TaPSTOL1-like* and *TaLrK10* within *Poaceae* species indicates that all species studied with this phylogeny belonged to three clades (Figure 2). The first clade consists of *OsPSTOL1* from *Oryzeae* species alone, suggesting the presence of *Oryzeae* species-specific haplotypes based on these two proteins (Figure S4; e.g., “YKGELPNGVPVAVKMLEN” in *Oryzeae* species versus “YRGSLPNGREIAVKMLKD” in *T. aestivum*_*Lr10K_* AAC49629_U51330). This clade clustered distantly from the second clade (see below). For instance, *OsPSTOL1* from *O. sativa* and *TaLrK10* from *T. aestivum* have 70% polymorphisms. Hence, *OsPSTOL1* from *Oryzae* species clustered distantly from those of *TaPSTOL1*/*TaLrK10* (clade II) of other tribes of *Poaceae* species.

The second clade comprises both *Triticeae* and other tribes of the *Poaceae* members due to a higher similarity between the aligned regions in the two proteins. For instance, “YRGDLSDGRQIAVKMLKD” from *TaPSTOL1*-3DL of bread wheat and “YRGGLSDGRQIAVKMLKD” from *AtLrK10* of *A. tauschii* differed by only 5.55% (Figure S4). On the other hand, a lower distance between *TaPSTOL1*/*TaLrK10*, for instance, *LrK10* from *O.brachyantha* and *TaPSTOL1*-3DL from *T. aestivum* (19% variation) clustered together in clade II. In clade III, only *TaLrK10* from *T. aestivum* appeared, which indicates that *TaPSTOL1* is distant to *TaLrK10* in bread wheat, probably due to the absence of the amino-terminal domain in *TaPSTOL1*. Further, this clade also has the *TaLrK10* sequence from chromosome 1AS (Genbank accession: Nucleotide_U51330, Protein_AAC49629). Therefore, the pattern of *TaLrK10* and *TaPSTOL1* gene divergences among the species studied appears to indicate two broad groups: while species in clade II have both *TaLrK10* (e.g., *RLKs*) and *TaPSTOL1* (e.g., *RLCKs*), the species in clade III appear to have limited to only *RLKs*. The overall pairwise mean distance through the JTT matrix-based model [27] suggests that both these proteins likely shared a common ancestral lineage. Overall, the polymorphic sites analysis revealed that *TaPSTOL1-like* has a higher nucleotide diversity compared to *TaLrK10* within *Triticeae*. However, it was unclear among the different tribe members of the *Poaceae* family (Table S3).

The homogenous substitution through Monte Carlo 1000 replicates-based Disparity Index test (I_D_ Test) indicates that 12.38% of sites evolved with a pattern of homogenous substitutions with a significant I_D_ Test. The estimated frequencies of amino acids for both genes ranged from 1.43% (W) to 9.11% (L). Substitution rate with 35 randomly selected sites (of 241 positions from 105 amino acid sequences), revealed that there are 13 conservative (C), ten semi-conservative (SC), and 12 non-conservative (NC) substitutions with the frequencies of 37.14%, 28.57%, and 34.29%, respectively. The sum of C and SC substitutions was 65.71%, which is higher than NC substitutions, suggesting that both genes share an evolutionary pattern of identity. These results indicate that both genes share a significant amount of sequence identity and hence are closely related to each other (Table S4).

**Figure 4:**
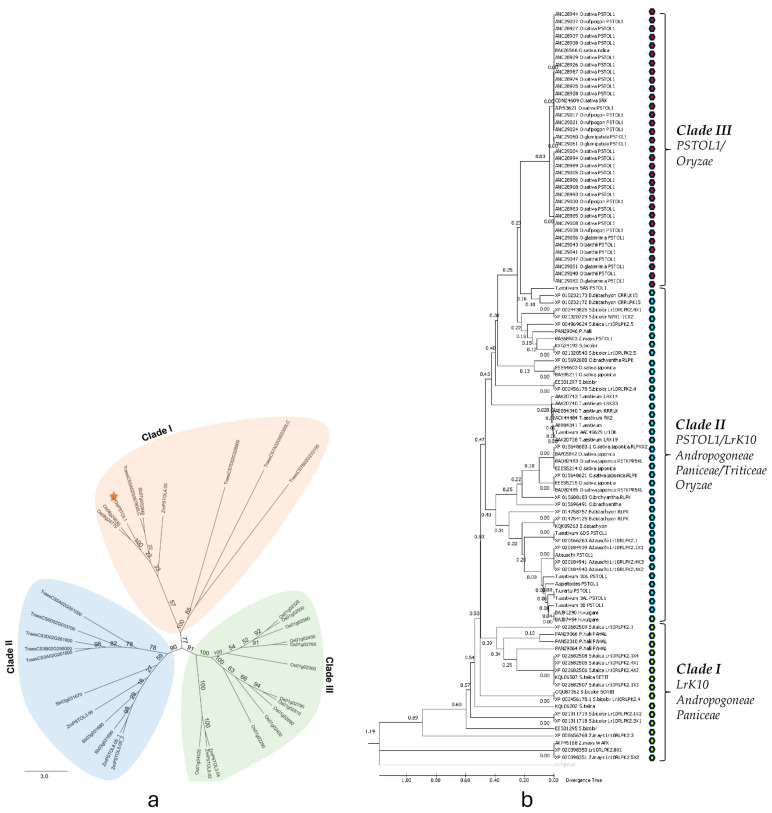
(**a**). Phylogenetic relationships of wheat, rice, sorghum and maize *PSTOL1*-like genes. An unrooted tree of six *PSTOL1*-like sequences from wheat, sorghum, maize and rice (four *PSTOL1* sequences and publicly available protein kinases) was included in phylogenetic analysis. Three distinct groups with three colours can be differentiated among these tribes’ members. Proteins marked with an asterisk is *OsPSTOL1* belongs to group II. (**b**). The relative divergence time tree showing the relative divergence of *PSTOL1* and *LrK10* among diverse Poaceae species and clustered groups among the tribes indicates the sequence domain-specific conservation across the tribes within Poaceae.

The protein sequences of *OsPSTOL1*, the nine *TaPSTOL1*-like proteins, and related kinases from specific species derived sequences such as rice, sorghum, and maize were used for phylogenetic analysis. The analysis revealed the presence of three distinct protein clades (Figure 4a). All wheat *TaPSTOL1*-like proteins clustered in two clades (I and II), which also included OsPSTOL1 and *PSTOL1*-like proteins from maize and sorghum. Clade I contained four *TaPSTOL1*-like proteins (TraesCS5A02G067800LC, TraesCS7A02G502300LC, TraesCS7B02G233100, TraesCS7D02G328900). From our analysis the closest OsPSTOL1 orthologs resulted to be TraesCS5A02G067800LC, ZmPSTOL4.05 and Sb07g002840. On the other hand, clade II contains the remaining five TaPSTOL1-like proteins (TraesCS3A02G261800, TraesCS3B02G295000, TraesCS3D02G261800, TraesCS5A02G051000 and TraesCS6D02G033700) along with three sorghum and maize orthologs. In clade II, there is a clear distinction between the proteins from sorghum, maize and wheat which reflects the two photosynthetically subtypes C4 and C3, respectively. By contrast, the majority of protein kinases within clade III derive from rice except for two *ZmPSTOL1*-like proteins. Overall, with the exception of TraesCS5A02G067800LC, the phylogenetic tree suggests a clear separation between *TaPSTOL1*-like proteins and the *PSTOL1*-like proteins from other grasses. Therefore, Figure 4a, with an unrooted tree, indicates the phylogenetic relationship among wheat, rice, sorghum and maize *PSTOL1*-like genes. However, the unrooted tree was generated using *PSTOL1*-like sequences alone rather than the combination of *PSTOL1* and *LrK10*; thus, it indicates a clear pattern of evolutionary relationships within these specific species concerning post-divergence-derived *PSTOL1* genes.

The protein-based divergence time tree indicated that the relative divergence time of *LrK10* from *Andropogoneae* and *Paniceae* species (Clade I) was earlier (0.57) (Figure 4b). Subsequently, *PSTOL1* and *LrK10* diverged (0.47) in *Triticeae*, *Oryzeae*, *Brachypodieae*, *Andropogoneae* and *Paniceae* species (Clade II) with similar divergence times. The Tajima relative substitution rate analysis also supports the presence of both *PSTOL1* and *LrK10* in clade II, as these two genes share numerous monomorphic sites (250 sites) (Table S5). Further analysis revealed a higher number of divergence sites and multiple sub-clusters comprising species involved in the divergence of both *PSTOL1*/*LrK10* from all four tribes. The *PSTOL1* most recently diverged in *Oryza* species (Clade III) (0.03). This analysis suggests that the environmental heterogeneity might have probably triggered a high divergence of *OsPSTOL1* in *Oryza species* [23]. This observation is also consistent with a lower pairwise nucleotide diversity noticed for *PSTOL1* (19%) compared to *LrK10* (30%). These results suggest that putative *PSTOL1* may have recently diverged compared to *LrK10* in the *Poaceae* tribe [9].

### 2.4. Putative PSTOL1-Related Kinase Search in Different Phyla

**Figure 5:**
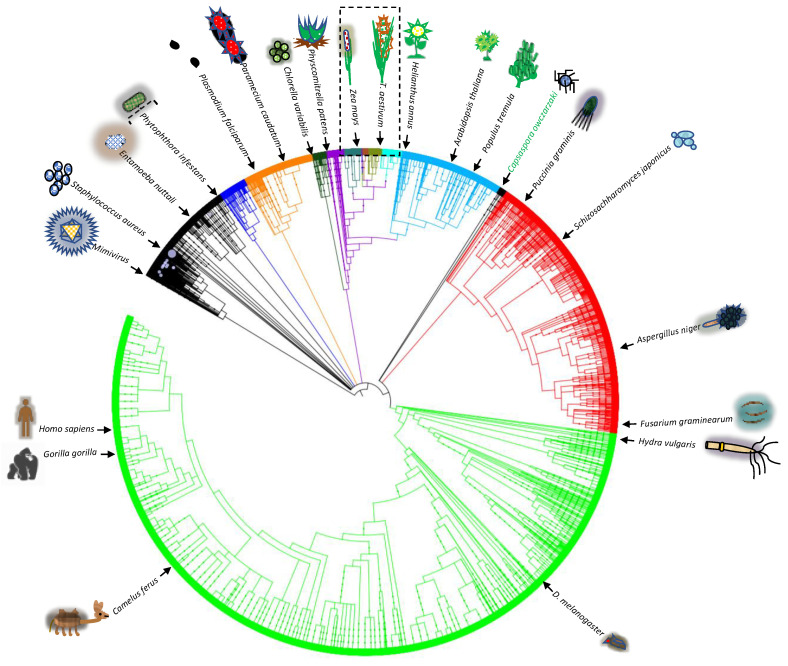
The life-tree is based on putative *PSTOL1*-related protein kinase sequences across several phyla. The section of Poaceae is shown in the dotted box. Orthologs of the putative *PSTOL1*-related protein kinase sequence from different phyla were predicted using the SMART (Simple Modular Architecture Research) tool, and the life tree was generated using iTOL. Each cluster with a different colour indicates the hierarchical structure with different phyla of the tree of life.

To understand the conservation of kinase-related genes across different phyla, a search for putative *PSTOL1* kinase-related protein sequences was performed in SMART, and the Lifetree-based representation is shown in Figure 5. This analysis indicates the versatile conservation of this kinase-related putative protein across different phyla represents the evolutionary importance of diversity and conservation of kinase-related genes.

### 2.5. Comparison of Putative TaPSTOL1 and TaLrK10 Protein Structures

We performed both motif and domain analyses for the protein-coding region of the putative *TaPSTOL1* of all homeologs in bread wheat, as well as the kinase-aligned region of AtLrK10 (kinase encoding part) from *A. tauschii*. The putative *TaPSTOL1*-3DL protein has 130 motifs, of which 57 were exclusively kinase-related, including three CDK (Cyclin-Dependent Kinase) motifs, one MAPK (Mitogen-activated protein kinase) motif, and one PIKK (Phosphoinositide-3-OH-kinase-related kinases) motif. The comparison between *PSTOL1* and *LrK10* among the *Triticeae*, *Oryzeae*, *Andropogoneae*, and *Brachypodeae* tribes revealed several conserved motifs, such as the CDK motif “RALIY” between positions 121–125; motif “RGLEY” between positions 162–166, except for the LrK10 in bread wheat; and the motif “KMLL” between positions 336–339. The putative *TaPSTOL1-3DL* from bread wheat showed a higher similarity with *AtLrK10* of *A. tauschii* in the kinase domain-encoding region. Genes belonging to the groups of RLCKs, including putative *TaPSTOL1*, do not possess the extracellular domain [19] but do maintain a predicted transmembrane domain and kinase domain (Figure S5).

We further studied the primary structural differences between putative *PSTOL1* and *LrK10* across several species of four tribes, which revealed the presence of 64 identical, 55 conservatively substituted and 12 semi-conservatively substituted residues. The kinase domain comprising 199 residues (starting at position 52 in *TaPSTOL1*-3DL in bread wheat) revealed that there are 54 identical, 39 conservative and seven semi-conservative replacements when aligned with *TaLrK10*. The ATP binding signature “LGQGGFGAVYRGDLSDGRQIAVKM” starting at position 59 was conserved between the two genes, with Gly17 conserved entirely across all the species studied. At the carboxyl (C) terminus, four identical and two conservative replacements were found between LrK10 and *PSTOL1* within the *Triticeae*, while the amino (N) terminal region was highly similar (Figure S5).

The predicted ATP binding signature of Tyrosine kinase comprises 57 residues, starting from position 86 and is highly conserved between *PSTOL1* and *LrK10* with 12 identical residues, 17 conservative and three semi-conservative changes. At the N-terminus, there were 23 residues entirely conserved between *AtLrK10* of *A. tauschii*, *B. distachyon*, and *TaPSTOL1*-6DS of bread wheat. About 12 residues within the Ser/Thr active site signatures (starting at position 175; “IVHFDIKPHNILL”) were highly conserved, with nine sites and three conservative amino acid replacements between *PSTOL1* and *LrK10*. Further analysis using either *TaPSTOL1*-3DL or *TaLrK10* protein as a query in the Classification of Protein Domain Structures (CATH) server resulted in the identification of the Ser/Thr receptor kinase *PR5K* (E-value: 1.1 × 10^−75^), which belongs to the group of phosphotransferase superfamily that catalyses the transfer of a phosphate to the hydroxyl groups of their protein substrates. We further superimposed 148 representative domains within the phosphotransferase superfamily, which indicated that this domain is present across several species (Figure S6).

**Figure 6:**
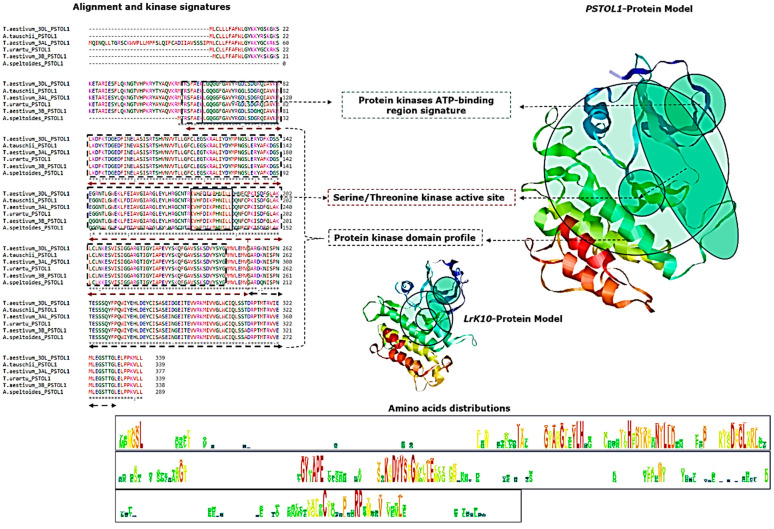
The protein 3D modelling of *PSTOL1* and *LrK10* and prediction of domains and aligned region of PSTOL1 within bread wheat and its progenitors.

The secondary structures of the putative *TaPSTOL1* and *TaLrK10* kinase domain regions are quite similar (Figure 6). The putative *TaPSTOL1* vary from 51 to 67 α-helices, while the kinase domain-aligned region of *TaLrK10* had 39 α-helices between *T. aestivum* and *A. tauschii*, respectively. These regions also exhibited a higher frequency of identical amino acids, which is also consistently observed for extended beta-strands and random coils. The isoelectric focusing point (pI) varied from six to nine between the two genes when compared to the homeologous sequences. The content of positively and negatively charged residues was similar between the two genes. The instability index estimated was 40 for both *TaPSTOL1* and the kinase domain-aligned region of *TaLrK10*. However, the instability index exceeded 40 when considering *TaLrK10* alone, which therefore appears to have a less stable protein compared to the putative *TaPSTOL1* (Table S6). Although less stable compared to the *PSTOL1* gene, the *LrK10* has shown consistency in having the WAK domain. The biological implications include that the biotic stress response is meaningful to state its functional relevancy more in *LrK10*, while specific homeologs of the *PSTOL1* gene indicate its role in biotic stress response. Therefore, evolutionarily diverged paralogous or homeologous copies of WAK-related genes have undergone functional specialisation and contribute to responses to biotic or abiotic stresses, as well as to other biological processes that depend upon the activation of distinct signaling pathways. (Recently accessed relevant contents on 27 April 2026, [28,29,30]).

Protein-3D modelling revealed that the structural similarity in the kinase domain region between putative *TaPSTOL1* and *TaLrK10* is in accordance with the alignment of the two predicted proteins corresponding to their nucleotide sequence (Figure S6a). The *TaLrK10* model was created using the template *Brassinosteroid insensitive 1-associated receptor kinase 1* (PDB: 3ULZ.1), which showed 43.42% identity with the aligned kinase domain region.

**Figure 7:**
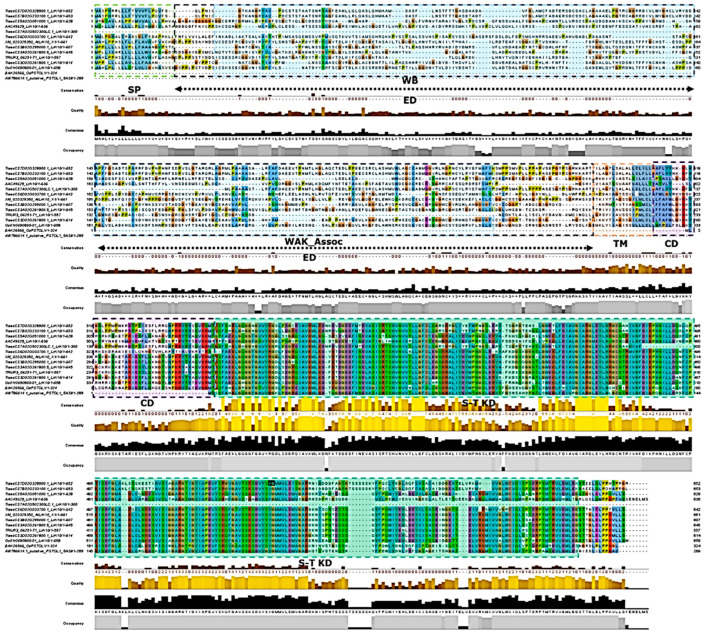
Protein domains, structural organisation and alignment between *LrK10* and *PSTOL1* gene orthologs and homeologs. SP: Signal Peptide, EB: Extra Cellular domain, TM: Transmembrane domain, CD: Conserved domain. S-T KD: Serine Threonine kinase domain. Domain predictions based on the source: [18].

The pattern of domains organisation of putative *TaPSTOL1* homeologs within *Triticum* species (Figure 7) suggests that it is similar to the *OsPSTOL1* of rice, in that it does not bear the extracellular domain, which is typically found in *RLCKs* [19,31]. In contrast, the putative *LrK10* from *A. tauschii* and *T. aestivum* have this extracellular domain [32]. We further observed enormous structural rearrangements in the upstream region between putative paralogs *TaPSTOL1*_*5ASII* and *TaPSTOL1_5ASI* (*TaLrK10_5ASI*). A comparison between *LrK10* and *PSTOL1* homeologs in wheat reveals the presence of two patterns of structural organisation, with either a single exon1 CDS (putative *PSTOL-like* gene) or three to four exons with a single or multiple transcripts with alternative splicing.

**Figure 8:**
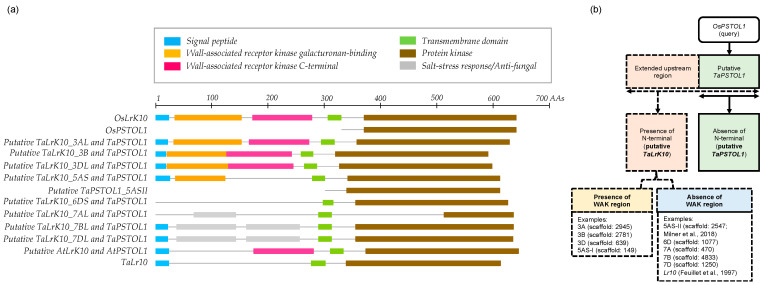
Structural organisation of putative *TaLrK10* and *TaPSTOL1* genes in Triticum species. (**a**) The organisation, presence, and absence of different domains in putative *TaLrK10* and *TaPSTOL1* in Triticum species. The amino-terminal domain is present in putative *TaLrK10* but absent in TaPSTOL1. Similarly, the WAK (wall-associated kinase) is observed in putative *LrK10* in *A. tauschii*, and a few homeologous putative *TaPSTOL1* in bread wheat, while absent in all other homeologs. (**b**) Schematic representation of putative *TaLrK10* and *TaPSTOL1*-like genes, and the presence or absence of the N-terminal domain and WAK region in wheat.

We noticed two distinct structural organisations for *TaLrK10* in *Triticum* species (Figure 8). In the wheat progenitor *A. tauschii*, *AtLrK10* (Genbank accession: XP_020184939) carries a partial *wall-associated protein kinase* carboxyl region (*WAK*; 94 amino acids) in the N-terminus, which is comparable across diverse *Poaceae* species, including sorghum [14]. However, the *WAK* domain appears to be absent in some of the *TaLrK10* homeologs in bread wheat (chromosomes 6 and 7), including *TaLr10* (Genbank accession: U51330) [18]. To better understand the organisation of domains in putative *PSTOL1* gene homeologs and orthologs, we have further depicted an illustration based on the prediction model of *LrK10* [18]. The alignment-based prediction model revealed the higher conservation of the Serine-Threonine Kinase domain in *PSTOL1* and its related *LrK10* protein.

**Figure 9:**
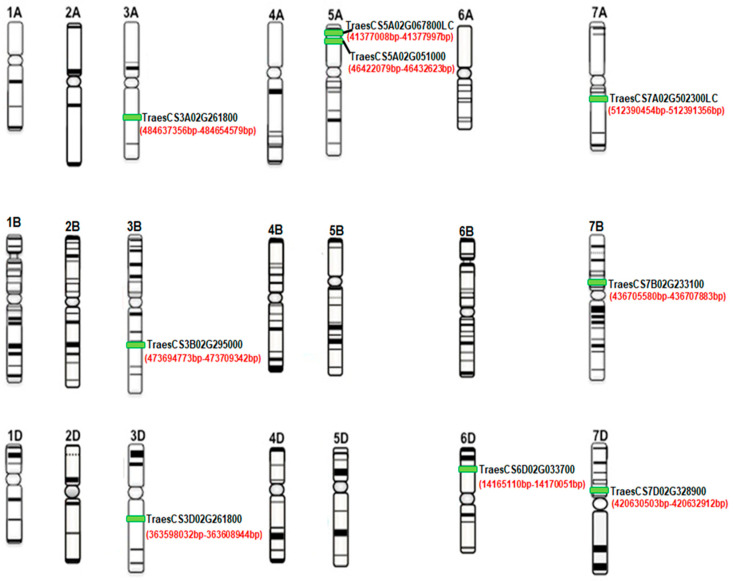
Distribution of *TaPSTOL* genes across the seven chromosome groups and A, B and D subgenomes of bread wheat. Genes are represented by green bars. Figure adapted from the IWGSC website (Accessed on 5 June 2020, http://www.wheatgenome.org/).

Figure 8 clearly reveals the presence and absence of specific domains from *TaLrK10* and *TaPSTOL1* genes. The presence of WAK (wall-associated kinase) is specific to *LrK10*, and some homeologs of *PSTOL1* also show its presence. However, some homeologs of *PSTOL1* lack the WAK domain, indicating the importance of WAK is more in the LrK10 gene rather than in the *PSTOL1* gene. Figure 9 reveals the physical location of *PSTOL1* genes in different homoeologous chromosomes in bread wheat. BLASTN analysis revealed the presence of remnants across different chromosomes, while the intact *PSTOL1* gene with structural organisation is known through domain patterns and physical locations.

## 3. Discussion

### 3.1. Commonalities and Dissimilarities of Putative TaPSTOL1-like and TaLrK10 Gene Sequences

Both *OsPSTOL1* and *TaLrK10* genes have been known to play different functional roles in plants. However, it is unknown whether they belong to the same ancestral lineage. Using a comparative bioinformatic framework, we find that both the putative *TaPSTOL1-like* and *TaLrK10* genes are closely related to each other, likely derived from a common ancestral origin. Based on BLASTP (2.17.0) and BLASTN, eight putative homeologs of *TaPSTOL1-like* were detected in bread wheat, consistent with several *PSTOL1* homologs/orthologs identified in other species such as sorghum (14) and maize [15]. Similar to *OsPSTOL1*, all eight putative wheat *TaPSTOL1*-like proteins are predicted to have a conserved Ser/Thr kinase domain. In contrast to our study, a recent study reported a single copy of *TaPSTOL1* on chromosome 5A in wheat [16]. Such a difference in the number of homeologs identified between these two studies could potentially be related to the incomplete (as well as continuously updated) nature of the wheat genome database and germplasm used.

While the putative *TaPSTOL1-like* homeologs identified based on the BLASTN at Wheat Ensembl analysis in our study were not functionally tested, we further leveraged the wheat expression database [24] to show that all the putative *TaPSTOL1-like* homeologs identified in our study showed enhanced expression under low-P as compared to control conditions, suggesting that all these putative homeologs could be expressed under low-P. Consistently, the expression of the *TaPSTOL1*-like gene was observed (on chromosome 5AS; Acc# MH043199) in root tips and root hairs under different P concentrations [16]. The putative *TaPSTOL1-like* homeologs identified in this study could therefore be analogous to previously reported *PSTOL1* genes in maize [14,15] and rice [9,11,12,13,23].

It is interesting to note that the conserved kinase region appears to have a high nucleotide similarity (96%) with the third exonic region of *TaLrK10_3DL* from bread wheat. The multiple-sequence alignment (*TaLrK10_3DL* and *TaPSTOL1-3DL*) further indicated that the putative *TaPSTOL1_3DL* overlaps with the genomic locations of *TaLrK10_3DL*. Indeed, the putative *TaPSTOL1* appears to have derived from the last exonic region (3rd exon) of *TaLrK10_3DL* in bread wheat, which indeed showed more than 70% of deletions relative to its ancestral *AtLrK10* from *A. tauschii*. While the putative *TaPSTOL1_3DL* appears to lack the other exons that encode the extracellular domain in *TaLrK10_3DL*, as was also observed in rice [9], these comparative analyses inform that the third exon of *TaLrK10_3DL* (3DL: 363,607,922 bp to 363,608,939 bp) would indeed act as a putative single exon-based *TaPSTOL1-like* gene in bread wheat. Therefore, the putative *TaPSTOL1* (*RLCK*) seems to have the variant of typical *TaLrK10* in bread wheat that belongs to the *RLK* family [33].

### 3.2. Both PSTOL1 and LrK10 Have Conserved Kinase Domains

The protein kinase family is a large, dynamic group with highly conserved functional domains and motifs [34,35]. Hence, we focused our comparative analysis on the relevance of the different structural organisation of domains and motifs. For instance, the motif “RAILY” is highly conserved between *PSTOL1* and *LrK10* across diverse species within the *Poaceae*. There were, however, some subtle differences detected at either the N-terminal or the C-terminal level and also observed for the RGLEY and KMLL motifs. The kinase domain was highly conserved with many identical amino acids, and the ATP-binding signature had 23 out of 24 amino acids highly conserved, indicating a robust structural redundancy in both the analysed proteins.

### 3.3. Structural Analysis of TaLrK10 Indicates Two Distinct Patterns in Triticum Species

The predicted physicochemical properties indicate the secondary and tertiary structures of the kinase domains of putative *TaPSTOL1-like* and *TaLrK10* are similar in the theoretical isoelectric point and instability index. It has previously been shown that an in vitro phosphorylation activity for *OsPSTOL1* using recombinant technology [9]. Consistent with this study, our computational analysis also indicates that *TaPSTOL1* and *TaLrK10* are closely related to the superfamily transferase (*Phosphotransferase*) domain 1 and are also closely associated with nonspecific *serine-threonine protein kinases* (2.7.11.1) and *serine-threonine protein kinases* (2.7.10.1), which are part of the CATH superfamily cluster number 12. Our analysis, therefore, indicates that the putative *TaPSTOL1-like* is structurally more similar to *AtLrK10-L2* of *A. tauschii* than to *TaLrK10* of *T. aestivum*.

We observed two distinct structural organisations for putative *TaLrK10* within *Triticum* species. *TaLrK10* contains a wall-associated kinase (*WAK*) carboxyl region at the C-terminus in the wheat progenitor *A. tauschii*, as was also predicted in some *SbPSTOL* homologs in sorghum [14]. However, such a *WAK* region is absent in the majority of the putative *TaLrK10* homeologs predicted in bread wheat. The *WAK* proteins possess a typical cytoplasmic Ser/Thr kinase signature, which typically carries an extracellular domain (e.g., the *LrK10* protein), mediating crosstalk with environmental signals [14,36,37]. It is known that chitin from fungal pathogens [38,39] and pathogen-based induction of salicylic acid trigger *WAK*-containing genes for the survival of host plants [40]. The apparent absence of such a *WAK* region in the majority of the putative *TaLrK10* homeologs (particularly *TaLr10* from 1A; [18] in bread wheat) supports the prevailing notion that the *TaLrK10* gene is no longer considered to be the ortholog of the *Lr10* gene in bread wheat, although it may still encode a kinase domain involved in plant-pathogen interactions [41]. We suspect that *TaLrK10*, lacking the *WAK* region, might have served as the basis for the divergence of the putative *TaPSTOL1-like* gene as a single-exon-based gene without the *WAK* region while retaining the kinase domain. The putative *TaPSTOL1-like*, which lacks the amino-terminal domain and thus is structurally similar to the third exon of *TaLrK10* in bread wheat. Further research is needed to validate the presence or absence of the *WAK* region, likely associated with different functional roles of RLK proteins in plants.

## 4. Material and Methods

### 4.1. Putative TaPSTOL1 and TaLrK10 Gene Sequences from Poaceae Members

We initially used the rice *OsPSTOL1* gene sequence (Accession ID: KU922625) [23] as a reference to identify close orthologs in the bread wheat cv. Chinese Spring reference genome [42,43]. Based on this query, we found top hit gene models TraesCS3A02G261800, TraesCS3B02G295000, and TraesCS3D02G261800 from group 3 chromosomes of wheat, which we further used for the 3DL ortholog (Accessed on 20 January 2019, contig: IWGSC_V3_chr3DL_scaffold_639, derived through rice *OsPSTOL1* using the URGI-IWGSC wheat BLASTN server https://wheat-urgi.versailles.inra.fr/Seq-Repository/BLAST) due to nascent polyploidization (AABB + DD) of the D genome to identify other homeologs across all chromosomes of bread wheat and named them as putative *TaPSTOL1-like* gene sequences. The presence of the kinase domain in these sequences was then predicted using Pfam Sequence Search [44] and ScanProsite (Accessed on 15 March 2019, https://prosite.expasy.org/scanprosite/).

Further, using the 3DL sequence as a reference, we searched for homologous/orthologous sequences from wheat wild progenitors such as *T. urartu*, *A. speltoides* and *A. tauschii* and members of the tribes of *Triticeae*, *Brachypoideae*, *Paniceae* and *Andropogoneae* using different public databases such as NCBI (Accessed on 11 January 2019, https://blast.ncbi.nlm.nih.gov, https://www.ncbi.nlm.nih.gov/datasets/docs/v2/citing-datasets/), URGI (Accessed on 22 March 2019 https://urgi.versailles.inra.fr, https://urgi.versailles.inrae.fr/Platform/How-to-cite) and *Aegilops tauschii* (The previously accessed *A. tauschii* genome database from UC-DAVIS is currently inactive, so we have recently accessed an alternative database: https://plants.ensembl.org/Aegilops_tauschii/Info/Index, https://asia.ensembl.org/info/about/publications.html, accessed on 28 April 2026). Query coverage of sequences with more than 70% identity was retrieved using BLASTN [45] with E-values 0 to 1 × 10^−130^. In total, 108 DNA sequences from different *Poaceae* species were aligned together using ClustalX [46].

### 4.2. Evolutionary Analysis of Putative TaPSTOL1 Gene Sequences

A phylogenetic tree was generated using the neighbour-joining method [47] with 1000 bootstrap resamplings [48]. The evolutionary distance was estimated using the maximum composite likelihood method [49]. The disparity index [50] using a Monte Carlo test with 500 replicates was estimated using MEGA 7 [51]. The Gamma statistic for gene flow estimates of haplotypes [52,53], Nst [54], Fst [55], synonymous to non-synonymous substitutions [56] and other population genetics analyses were performed using DNAsP V5 [57]. The genomic to mRNA alignment was performed using SPLIGN [58]. The prediction of MITE (Miniature Inverted Repeat Transposable Element) terminal inverted repeat was performed using the EMBOSS inverted repeat server, and target site duplication was manually assessed. The miRNA prediction was performed in miRBase [59], and target genes for the miRNA were predicted using psRNATarget [60]. Promoter motif prediction was performed using the PLACE server [61].

### 4.3. Evolutionary Analysis of Putative TaPSTOL1 and TaLrK10 Protein Sequences

Ortholog and paralog sequences of the putative *TaPSTOL1* protein from diverse species belonging to the tribes of *Triticeae*, *Brachypoideae*, *Paniceae*, and *Andropogoneae* (with a query coverage > 70%, identity > 85%, and E-value: 0 to 3 × 10^−150^) were retrieved from NCBI. The sequences were aligned using ClustalX using default parameters [46]. Subsequently, a phylogenetic tree using the neighbour-joining method [47] with 1000 bootstrap resamplings [48], evolutionary distance using the Jones-Taylor-Thorns (JTT) method [62], a RelTime relative divergence tree [63], a Tajima neutral evolutionary and relative rate test [64], rates of amino acid substitutions using the JTT [27] model through the maximum log-likelihood method, and a disparity index [50] were generated in MEGA7 [51].

### 4.4. Retrieval of Protein PSTOL1 and LrK10 Gene/Protein Sequences in Wheat and Close Relatives

Phylogenetic analysis of the nine *TaPSTOL1*-like protein sequences was performed in Geneious^®^ 8.1.3 using the Geneious plugin PhyML (Accessed on 12 May 2021, http://www.atgc-montpellier.fr/phyml). Published protein sequences of rice *OsPSTOL1* (AB458444.1), fourteen rice protein kinases (*Os01g02790.1*, *Os01g02810.1*, *Os01g02690.1*, *Os01g02400.1*, *Os01g02290.1*, *Os01g02320.1*, *Os01g04520.1*, *Os01g02550.1*, *Os01g02580.1*, *Os01g02300.1*, *Os01g02430.1*, *Os01g02760.1*, *Os08g24770.1*, *Os08g24630.1*), four sorghum *SbPSTOL1* (*Sb07g002840*, *Sb03g031690*, *Sb03g031680* and *Sb03g031670*) and six maize *PSTOL1* (*ZmPSTOL3.04*, *ZmPSTOL3.06*, *ZmPSTOL4.05*, *ZmPSTOL8.02*, *ZmPSTOL8.05_1* and *ZmPSTOL8.05_2*) genes were included in phylogenetic analysis.

For a comparative genomic study, an ancestrally kinase-related *TaLrK10* gene from *T. aestivum* was retrieved from the NCBI nucleotide database (accession: U51330) [18] and was further subjected to BLASTP to retrieve the related sequences from *T. aestivum*. Further alignments with *TaPSTOL1-3DL* revealed that the *T. aestivum Kinase 2* (*receptor-like kinase 2*; Genbank accession: ACK44484) is closely related to the putative *TaPSTOL1* and was therefore included in phylogenetic and protein sequence analysis. Further, the BLASTP analysis of putative *TaPSTOL1-3DL* revealed the highest amino acid sequence similarity (100% query coverage and 96% identity) with putative *AtLrK10* isoforms X1, X2 and X3 from *A. tauschii*.

### 4.5. Protein 3D Modelling and Annotations

Protein 3D modelling was performed using Geno3D [65], and annotation and visualisation were carried out using the RASMOL Viewer [66]. Kinase domains and active signatures were predicted using ScanProsite tools, and the documentation of protein physicochemical parameters, including instability index, was calculated using the Protparam tool using default parameters at Expasy (Accessed on 20 January 2019, https://web.expasy.org) [67]. Protein motifs were predicted using the Eukaryotic Linear Motif (ELM) resource [68]. Functional similarities with the existing X-ray crystal-based PDB structures (5LVO), gene ontology and superfamily superposition were predicted and annotated using the CATH database [69]. Outlier homologs of known structure, PFAM domains, signal peptides, and internal repeats were predicted using SMART (Simple Modular Architecture Research) [70] and a Lifetree was generated using iTOL [71]. Dot plot representation was performed using the EMBOSS online server [72].

### 4.6. Expression of Putative TaPSTOL1 Homeologous Sequences

We obtained the gene expression data of putative *TaPSTOL1* homeologs from the wheat expression database (Accessed on 23 February 2019 http://www.wheat-expression.com), which was already published [24]. Briefly, 14-day-old Chinese spring seedlings (CS) were subjected to P-starvation for ten days, and root and shoot samples were collected at 0 days (control) and at ten days (P-starvation). Total RNA was extracted from all tissues, and sequencing was performed on each library to generate 100-bp paired-end reads for transcriptomic sequencing on an Illumina High-Seq 2000 platform [24]. We have downloaded the raw data from the wheat expression database, and mean expression (Transcripts per Million-TPM) was obtained for all the putative *TaPSTOL1* homeologs.

## 5. Conclusions

Within the comparative genomics framework, our study examines that both the putative *TaPSTOL1* and *TaLrK10* genes are evolutionarily related to each other. However, the divergence of *TaLrK10* occurred earlier than the putative *TaPSTOL1* evolved. Each gene appears to have slightly modified functional properties, such as the N-terminus and the extracellular domain, and there is an insertion of a new MITE in the 5′UTR of the putative *TaPSTOL1*. Gene structure-based sequence analysis revealed that *TaPSTOL1*-like genes might have diverged from and originated from TaLrK10, and the resemblance of the third exon of *TaLrK10* is highly supportive of this hypothesis. This indicates the possibility of a truncated receptor kinase, while being functionally conserved in *TaPSTOL1*, as that of *TaLrK10*. The shared properties of their amino acids indicate that most of the mutations between these two genes could be synonymous, and gene conversion supports the presence of tracts between these two genes among Poaceae species. Studying the evolutionary and functional aspects of the putative *TaPSTOL1* and *TaLrK10* through gene-knockout models would further facilitate the comprehensive understanding of their functional roles in plants. Overall, all analyses with a specific focus on an evolutionary aspect revealed possible evolutionary divergence of these genes driven by domain loss and specialization to acquire specific functional patterns.

## Data Availability

Appropriate database links, relevant citation links, and dates of access were provided for the sequences used through public repositories such as NCBI, Ensembl, URGI, etc.

## Supplementary Materials

The following supporting information can be downloaded at: https://www.mdpi.com/article/10.3390/ijms27104513/s1.
