# Supplementary material for: Computational Predictions and Evolutionary Analysis of LrK10 Kinase-Related Putative PSTOL1 Gene Homeologs in Wheat and Orthologs of Its Wild Relatives
Source: Int J Mol Sci. 2026 May 18;27(10):4513. doi: 10.3390/ijms27104513 (PMC13207577; doi:10.3390/ijms27104513)
Supplement: Supplementary file 1 [file ijms-27-04513-s001.zip › ijms-4239674-supplementary.pdf]

Supplementary Information

S1 Table: Identified orthologs/paralogs of *PSTOL1* gene in wheat wild progenitors and homeologs of durum wheat and bread wheat and contig IDs.

Description	NI%	PI%	PQC%	KD	IWGSC-URGI Contigs
**Tu Paralog1**	96	96	100	Present	TGAC_WGS_urartu_v1_contig_229783
**Tu Paralog2**	89	83	41	Present	TGAC_WGS_urartu_v1_contig_167187
**Tu Paralog3**	94	71	85	Present	TGAC_WGS_urartu_v1_contig_173408
**Tu Paralog4**	67	68	97	Present	TGAC_WGS_urartu_v1_contig_168678
**AsParalog1**	95	95	85	Present	TGAC_WGS_speltoides_v1_contig_196855
**AsParalog 2**	85	90	66	Present	TGAC_WGS_speltoides_v1_contig_205688
**AsParalog3**	94	90	60	Present	TGAC_WGS_speltoides_v1_contig_238548
**AsParalog4**	66	71	100	Present	TGAC_WGS_speltoides_v1_contig_588662
**AtParalog1**	96	96	100	Present	TGAC_WGS_tauschii_v1_contig_1015851
**AtParalog 2**	68	68	100	Present	TGAC_WGS_tauschii_v1_contig_94060
**TdParalog1**	96	93	100	Present	TGAC_WGS_strongfield_v1_contig_366404
**TdParalog 2**	93	94	79	Present	TGAC_WGS_strongfield_v1_contig_710944
**TdParalog 3**	95	96	70	Present	TGAC_WGS_strongfield_v1_contig_469195
**TdParalog 4**	84	83	100	Present	TGAC_WGS_strongfield_v1_contig_2169382
**TdParalog 5**	73	72	90	Present	TGAC_WGS_strongfield_v1_contig_5032935
**Homeolog_3DL**	100	100	100	Present	IWGSC_V3_chr3DL_scaffold_639
**Homeolog_3AL**	96	96	100	Present	IWGSC_V3_chr3AL_scaffold_2945
**Homeolog_3B**	96	96	100	Present	IWGSC_V3_chr3B_scaffold_2781
**Homeolog_6DS**	90	87	100	Present	IWGSC_V3_chr6DS_scaffold_1077
**Homeolog_5ASI**	84	81	85	Present	IWGSC_V3_chr5AS_scaffold_2547
**Homeolog_5ASII**	70	71	100	Present	IWGSC_V3_chr5AS_scaffold_149
**Homeolog_7DL**	70	68	100	Present	IWGSC_V3_chr7DL_scaffold_1250
**Homeolog_7AL**	69	65	67	Present	IWGSC V3 chr7AL, Scaffold: 470
**Homeolog_7BL**	69	66	97	Present	IWGSC_V3_chr7BL_scaffold_4833

Abbreviations: NI: Nucleotide Identity, PI: Protein Identity, PQC: Protein Query Coverage, KD: Kinase Domain.

S2 Table. MITE (Miniature Inverted Repeat Transposable Element) from 5’ UTR of wheat wild progenitors, durum wheat and bread wheat.

**Species**	Presence or absence of the *PSTOL1* gene	Presence or absence of the MITE at 5' UTR of this *PSTOL1* gene
T. urartu	Present	Present
A. speltoides	Present	Absent
A.tauschii	Present	Absent
T. monococcum	Present	Absent
A. sharonensis	Present	Absent
T. durum		
**Copy1_3AL ortholog**	Present	Present
**Copy2_3B Ortholog**	Present	Absent
T. aestivum		
**Copy1_ Chromosome 3AL**	Present	Present
**Copy2_Chromosome 3B**	Present	Absent
**Copy3_Chromosome 3DL**	Present	Present with many mutations

S3 Table. Parsimony Informative Sites (PIS) and other sites in each group of the genes from diverse species.

Group	S/S	PS	MS	SV	PIS	SV2V	PIS2V	SV3V	PIS3V	SV4V	PIS4V	P_LD	χ^2
*PSTOL1*_Triticeae	8/1139	316	544	259	57	241	46	18	10	0	1	1035	178
*LrK10*_Triticeae	16/1027	282	715	70	212	70	175	0	32	0	5	15225	4568
*PSTOL1*_All	57/1139	340	456	94	246	92	178	3	61	0	7	36046	24012
*LrK10*_All	51/1043	450	310	60	390	56	199	3	129	1	62	29890	7557

Legend: S/S: Number sequences/Number of sites analyzed. PS: Parsimony Informative Sites (PIS), MS: Monomorphic Sites (MS), SV: Singleton Variable Sites, SV2V: Singleton Variable Sites with Two Variants (SV2V), PIS2V: Parsimony Informative Sites with Two Variants, SV3V: Singleton Variable with Two Variants, PIS3V: Parsimony Informative sites with Three Variants, SV4V: Singleton Variable with Two Variants, PIS4V: Parsimony Informative sites with Three Variants, LD: Parsimony Informative sites in LD derived from pairwise comparison, $\chi 2$-Number of Chi-Square significance of the compared sites in LD.

S4 Table. Randomly selected amino acids to assess type of substitutions from Maximum Likelihood Estimate of Substitution Matrix. C: Conservative substitutions, SC-Semi-conservative substitutions, NC: Non-conservative substitutions.

**Original**	**Substitution Amino acid**				
**Amino acid**	G	P	V	S	T
**Alanine**	0.69	0.78	1.16	1.55	1.83
**Type**	SC	NC	SC	C	NC
**Amino acid**	W	G	Q	H	K
**Arginine**	0.33	0.36	0.80	0.85	1.73
**Type**	NC	NC	SC	SC	SC
**Amino acid**	T	K	H	S	D
**Asparagine**	0.52	0.56	0.89	1.12	1.22
**Type**	SC	C	C	SC	C
**Amino acid**	A	H	G	N	E
**Aspartic acid**	0.22	0.27	0.34	1.48	2.07
**Type**	NC	SC	SC	C	C
**Amino acid**	H	R	W	Y	S
**Cysteine**	0.08	0.11	0.12	0.22	0.23
**Type**	NC	NC	NC	NC	SC
**Amino acid**	P	K	R	E	H
**Glutamine**	0.34	0.63	0.64	0.73	1.21
**Type**	NC	C	C	C	C
**Amino acid**	A	G	K	Q	D

S5 Table. Tajima Relative Test. Neutrality substitution rate was calculated for PSTOL1 using the taxas: A (*T. aestivum* 3DL-*PSTOL1*) and B (ANC28927 *O. sativa* PSTOL1) with sequence C (A. tauschii *PSTOL1*). For Lr10Kinase, A (ABB84341 *T. aestivum*) and B (XP 021311718 S. bicolor Lr10RLPK2.3X1), C (XP 020184941 *A.tauschii* Lr10RLPK2.4X3). For *PSTOL1*/*LrK10* Kinase genes A (*T. aestivum* 3DL-*PSTOL1*) and B (XP 014758757 *B. distachyon* RLPK), with sequence C (XP 020166263 *A. tauschii* Lr10RLPK2.1). The $\chi 2$ test statistic was 13.09 (P = 0.00030 with 1 degree of freedom). P-value less than 0.05 is often used to reject the null hypothesis of equal rates between lineages. The analysis carried out with MEGA 7, the analysis consists of 3 amino acid sequences.

Configuration	*PSTOL1*	*Lr10Kinase*	*PSTOL1/Lr10Kinase*
Identical sites in all three sequences	198	164	250
Divergent sites in all three sequences	7	44	9
Unique differences in Sequence A	2	107	10
Unique differences in Sequence B	81	11	34
Unique differences in Sequence C	1	5	22
P-value	0.0000	0.0000	0.0003
$\chi 2$	75.19	78.10	13.09
Degree of freedom	1	1	1

S6 Table. SPCP- structural and physico-chemical properties. NAA-Number of amino acids, AH- Alpha helix, ES- Extended strand, RC- Random coil, pI- Theoretical Isoelectric focusing point, NCR-Negatively charged residues (Aspartic acid and Glutamic acid), PCR-Positively charged residues (Arginine and Lysine), ID- Instability Index, AI- Aliphatic index. P3DL-*PSTOL1*:3DL, *LrK10*-Leaf Rust Resistance Kinase, *LrK10A*-Leaf Rust Resistance Kinase *PSTOL1* Aligned Region, P3AL-*PSTOL1*:3AL, P5AS-*PSTOL1*:5AS. PAt-*PSTOL1-A. tauchii*, *RK2-T. aestivum Receptor like kinase 2,* RK2A-*T. aestivum Receptor like kinase 2* aligned region.

SPCP	P3DL	LrK10	LrK10A	P3AL	P5AS	PAt	RK2	RK2A
**NAA**	339	636	350	377	289	339	634	350
**AH**	138	227	170	158	120	131	223	162
**ES**	59	104	39	63	51	67	108	43
**RC**	142	305	141	156	118	141	303	145
**pI**	8.52	6.34	8.23	8.67	6.24	8.67	8.53	9.07
**NCR**	38	69	38	38	33	37	61	35
**PCR**	42	64	40	44	31	42	69	42
**ID**	36.42	44.57	36.61	36.35	30.31	36.29	42.53	33.90
**AI**	86.25	87.22	91.89	89.97	81.97	87.96	87.93	92.14

S1 Fig.

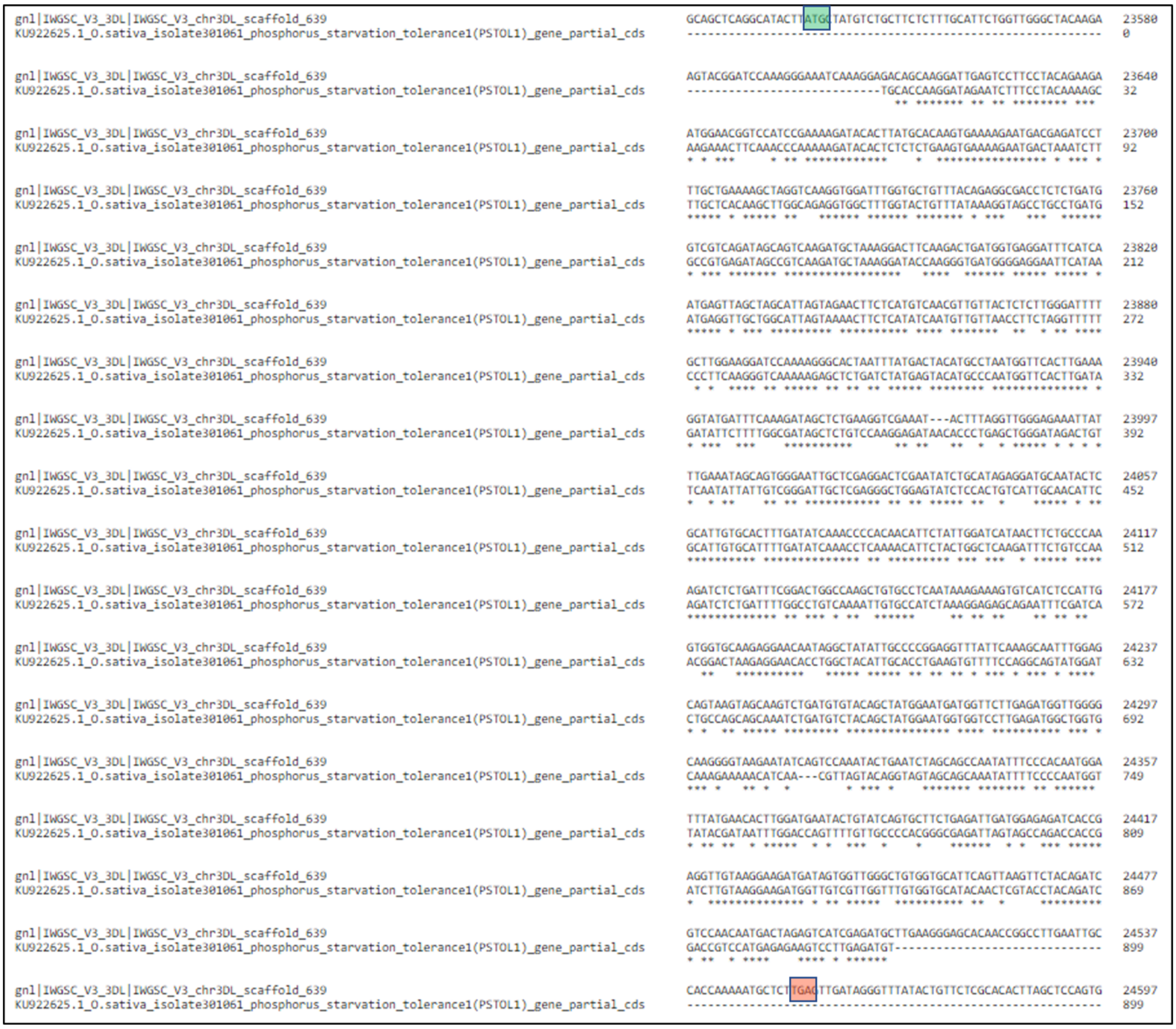

S1 Fig. Alignment between *O. sativa PSTOL1* gene and *PSTOL1* gene from 3DL chromosome of bread wheat.

S2 Fig.

(a)

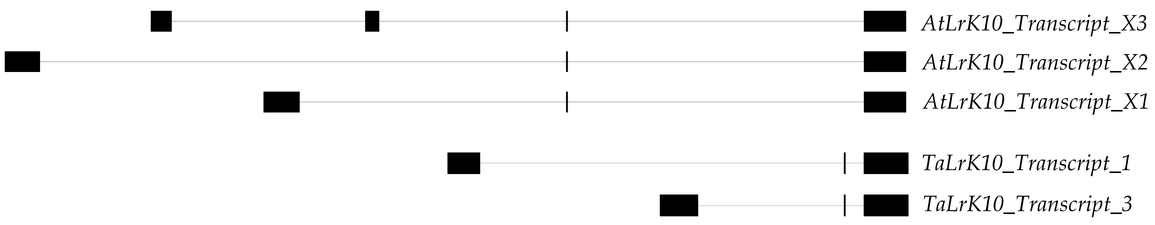

(b)

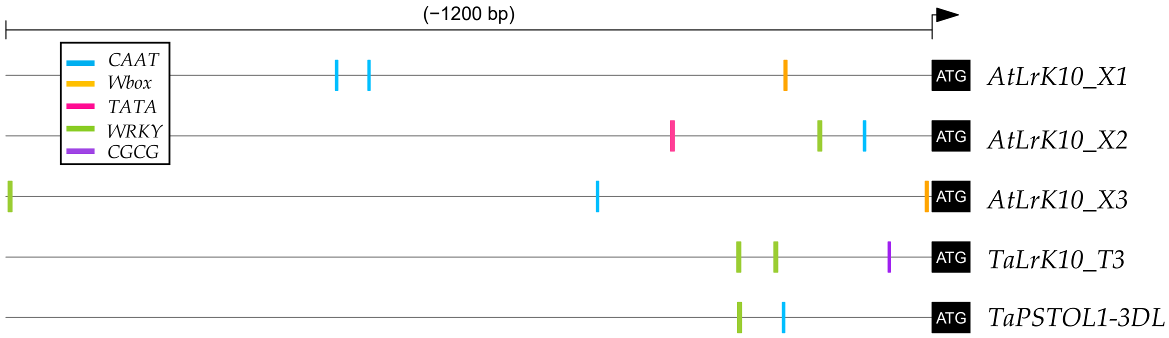

S2a Fig. Gene comparison between *LrK10* and *PSTOL1* in bread wheat and it's wild relative *Aegilops tauschii*. Different putative transcript variants of *LrK10* in *A.tauschii*, bread wheat and putative *PSTOL1-like* in bread wheat. Transcript variant X3 in *A.tauschii* has four predicted exons while all other transcripts in *A.tauschii* and bread wheat have three predicted exons. The conserved kinase domain across these multiple transcripts can be noticeable (the last exon in all transcripts). The black-filled rectangles indicate exons while horizontal grey-colored lines represent introns. **S3b Fig.** Predicted promoter transcription factor binding motifs in the upstream region (-1200 bp) for transcripts of *AtLrK10* and *TaLrK10* (Chromosome 3D).

S3 Fig.

```
>Chr3    Length=627182665

 Score = 1648 bits (892),  Expect = 0.0
 Identities = 976/1018 (96%), Gaps = 0/1018 (0%)
 Strand=Plus/Minus

Query  1          ATGCTATGTCTGCTTCTCTTTGCATTCTGGTTGGGCTACAAGAAGTACGGATCCAAAGGG  60
                  |||||||||||||||||||||||||||||||||||||||||||||||| |||||||||||
Sbjct  371108160  ATGCTATGTCTGCTTCTCTTTGCATTCTGGTTGGGCTACAAGAAGTACAGATCCAAAGGG  371108101

Query  61         AAATCAAAGGAGACAGCAAGGATTGAGTCCTTCCTACAGAAGAATGGAACGGTCCATCCG  120
                  |||||||||||||||||||||||||||||||||||||||||||||||||||||||||||
Sbjct  371108100  AAATCAAAGGAGACAGCAAGGATTGAGTCCTTCCTACAGAAGAATGGAACGGTCCATCCA  371108041

Query  121        AAAAGATACACTTATGCACAAGTGAAAAGAATGACGAGATCCTTTGCTGAAAAGCTAGGT  180
                  ||||||||||||||||||||||||||||||||||||||||| ||||||||||||||||||
Sbjct  371108040  AAAAGATACACTTATGCACAAGTGAAAAGAATGACGAGATCTTTTGCTGAAAAGCTAGGT  371107981

Query  181        CAAGGTGGATTTGGTGCTGTTTACAGAGGCGACCTCTCTGATGGTCGTCAGATAGCAGTC  240
                  ||||||||||||||||||||||||||||| | |||||| ||||| ||||||||||| ||
Sbjct  371107980  CAAGGTGGATTTGGTGCTGTTTACAGAGGTGGCCTCTCGGATGGCCGTCAGATAGCTGTG  371107921

Query  241        AAGATGCTAAAGGACTTCAAGACTGATGGTGAGGATTTCATCAATGAGTTAGCTAGCATT  300
                  |||||||||||||||| |||||||||||| |||||||||||||| ||| |||||||||||
Sbjct  371107920  AAGATGCTAAAGGACTACAAGACTGATGGGGAGGATTTCATCAACGAGGTAGCTAGCATT  371107861

Query  301        AGTAGAACTTCTCATGTCAACGTTGTTACTCTCTTGGGATTTTGCTTGGAAGGATCCAAA  360
                  ||||||||||||||||||||||||||||| ||||| ||||||||||||||||||||||||
Sbjct  371107860  AGTAGAACTTCTCATGTCAACGTTGTTACGCTCTTAGGATTTTGCTTGGAAGGATCCAAA  371107801

Query  361        AGGGCACTAATTTATGACTACATGCCTAATGGTTCACTTGAAAGGTATGATTTCAAAGAT  420
                  |||||||||||||||||||| |||||||||||||||||||||||||||| ||||||||||
Sbjct  371107800  AGGGCACTAATTTATGACTATATGCCTAATGGTTCACTTGAAAGGTATGCTTTCAAAGAT  371107741

Query  421        AGCTCTGAAGGTCGAAATACTTTAGGTTGGGAGAAATTATTTGAAATAGCAGTGGGAATT  480
                   ||||||||||| |||| | ||| |||||||||||||||||||| |||||||||||||||
Sbjct  371107740  GGCTCTGAAGGTGGAAACATTTTGGGTTGGGAGAAATTATTTGATATAGCAGTGGGAATT  371107681

Query  481        GCTCGAGGACTCGAATATCTGCATAGAGGATGCAATACTCGCATTGTGCACTTTGATATC  540
                  |||||||||||||||||||| || ||||||||||| |||||||| ||||| |||||||||
Sbjct  371107680  GCTCGAGGACTCGAATATCTCCACAGAGGATGCAACACTCGCATAGTGCATTTTGATATC  371107621

Query  541        AAACCCCACAACATTCTATTGGATCATAACTTCTGCCCAAAGATCTCTGATTTCGGACTG  600
                  || ||||||||||| || |||||||| |||||||| |||||||||||||||||||| |||
Sbjct  371107620  AAGCCCCACAACATCCTGTTGGATCAAAACTTCTGTCCAAAGATCTCTGATTTCGGGCTG  371107561

Query  601        GCCAAGCTGTGCCTCAATAAAGAAAGTGTCATCTCCATTGGTGGTGCAAGAGGAACAATA  660
                  ||||||||||||||||||||||||||||||||||||||||||||||||||||||||||||
Sbjct  371107560  GCCAAGCTGTGCCTCAATAAAGAAAGTGTCATCTCCATTGGTGGTGCAAGAGGAACAATA  371107501

Query  661        GGCTATATTGCCCCGGAGGTTTATTCAAAGCAATTTGGAGCAGTAAGTAGCAAGTCTGAT  720
                  ||||| |||||||| |||||||| || |||||||||||||||||||||||||||||||||
Sbjct  371107500  GGCTACATTGCCCCCGAGGTTTACTCGAAGCAATTTGGAGCAGTAAGTAGCAAGTCTGAT  371107441

Query  721        GTGTACAGCTATGGAATGATGGTTCTTGAGATGGTTGGGGCAAGGGGTAAGAATATCAGT  780
                  |||||||||||||||||||||||||||||||||||||||||||||| |||||||||||||
Sbjct  371107440  GTGTACAGCTATGGAATGATGGTTCTTGAGATGGTTGGGGCAAGGGATAAGAATATCAGT  371107381

Query  781        CCAAATACTGAATCTAGCAGCCAATATTTCCCACAATGGATTTATGAACACTTGGATGAA  840
                  ||||| ||||||||||||||||||||||||||||||||||||||||||||||||||||||
Sbjct  371107380  CCAAACACTGAATCTAGCAGCCAATATTTCCCACAATGGATTTATGAACACTTGGATGAA  371107321

Query  841        TACTGTATCAGTGCTTCTGAGATTGATGGAGAGATCACCGAGGTTGTAAGGAAGATGATA  900
                  ||||||||||||||||||||||||||||||||||||||||||||||||||||||||||||
Sbjct  371107320  TACTGTATCAGTGCTTCTGAGATTGATGGAGAGATCACCGAGGTTGTAAGGAAGATGATA  371107261

Query  901        GTGGTTGGGCTGTGGTGCATTCAGTTAAGTTCTACAGATCGTCCAACAATGACTAGAGTC  960
                  ||||||||||||||||||||||||||||||||||||||||||||||||||||||||||||
Sbjct  371107260  GTGGTTGGGCTGTGGTGCATTCAGTTAAGTTCTACAGATCGTCCAACAATGACTAGAGTC  371107201

Query  961        ATCGAGATGCTTGAAGGGAGCACAACCGGCCTTGAATTGCCACCAAAAATGCTCTTGA  1018
                   ||||||||||||||||||||||||||||||||||||||||||||||| |||||||||
Sbjct  371107200  GTCGAGATGCTTGAAGGGAGCACAACCGGCCTTGAATTGCCACCAAAAGTGCTCTTGA  371107143
```

S3 Fig. *PSTOL1* alignment from chromosome group 3 of *A. tauschii* and *PSTOL1*-3DL from bread wheat.

S4 Fig.

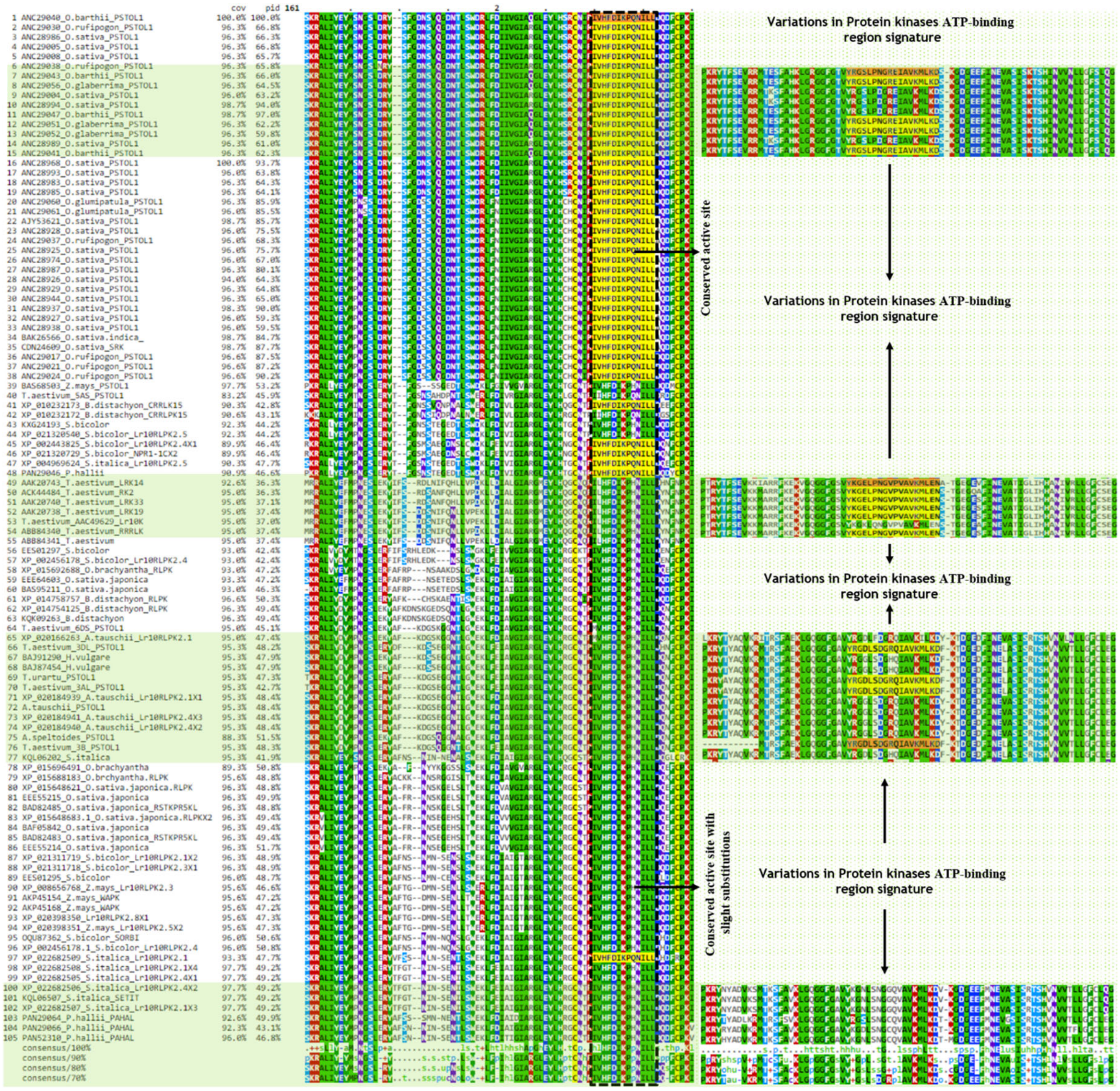

S4 Fig. Alignment showing similarities among the conserved region alignment from different species of *poaceae* between *PSTOL1* gene and *LrK10* gene members.

S5 Fig.

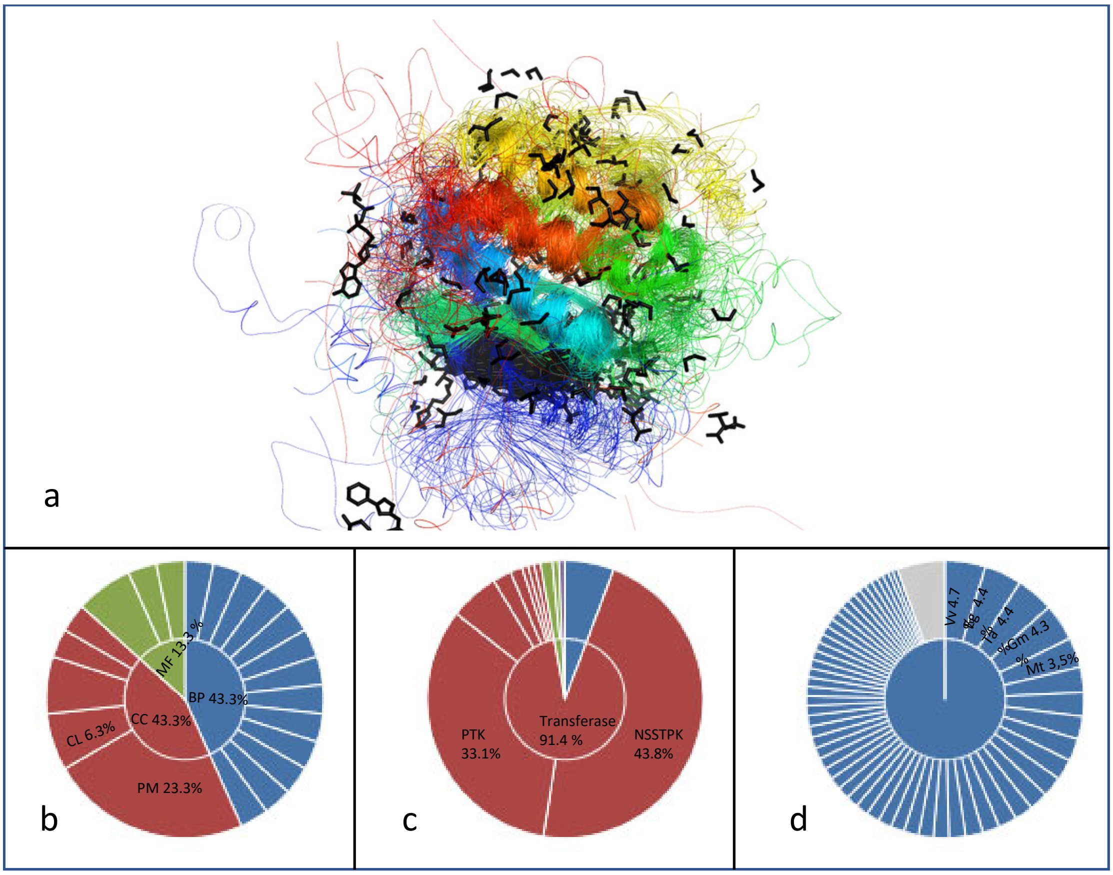

S5 Fig. Superposition of 148 representative kinase domains within the superfamily Transferase (Phosphotransferase) domain 1 (a); Gene ontology indicating the percentage of functional properties of this superfamily (b); Enzyme commission numbers showing the percentage of non-specific serine/threonine protein kinase within this superfamily (c); and observation of the percentage of species diversity concerning this superfamily (d). BP: Biological Process, CL: Cytosol, CC: Cellular Components, MF: Molecular Function.

S6 Fig.

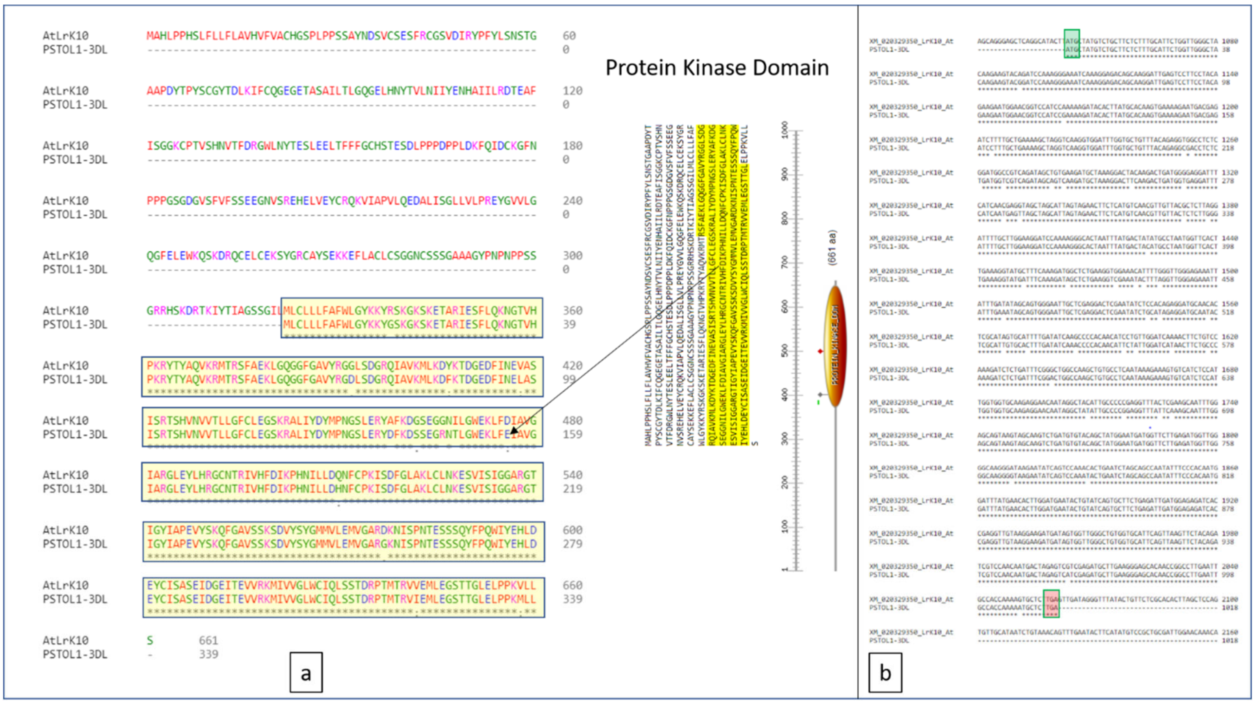

S6 Fig. (a) The protein alignment of *PSTOL1*-3DL from bread wheat and *LrK10* gene from *A. tauschii* on their kinase domain. (b) Aligned regions of nucleotide sequences covering region of *PSTOL1* gene with *LrK10* gene.
